# Increased abundance of *Ruminococcus gnavus* in gut microbiota is associated with moyamoya disease and non-moyamoya intracranial large artery disease

**DOI:** 10.1038/s41598-022-24496-9

**Published:** 2022-11-24

**Authors:** Yohei Mineharu, Yasuhisa Nakamura, Noriaki Sato, Takahiko Kamata, Yuki Oichi, Tomoko Fujitani, Takeshi Funaki, Yasushi Okuno, Susumu Miyamoto, Akio Koizumi, Kouji H. Harada

**Affiliations:** 1 Department of Neurosurgery, Kyoto University Graduate School of Medicine, Kyoto, Japan; 2 Department of Artificial Intelligence in Healthcare and Medicine, Kyoto University Graduate School of Medicine, 54 Shogoin Kawahara-Cho, Sakyo, Kyoto, 606-8507 Japan; 3 Department of Health and Environmental Sciences, Kyoto University Graduate School of Medicine, Yoshida Konoe-cho, Sakyo, Kyoto, 606-8501 Japan; 4 Department of Biomedical Data Intelligence, Kyoto University Graduate School of Medicine, Kyoto, Japan; 5 Social Health Medicine Welfare Laboratory, Public Interest Incorporated Association Kyoto Hokenkai, Kyoto, Japan

**Keywords:** Stroke, Cerebrovascular disorders, Diagnostic markers

## Abstract

Moyamoya disease (MMD) is a rare cerebrovascular disease endemic in East Asia. The p.R4810K mutation in *RNF213* gene confers a risk of MMD, but other factors remain largely unknown. We tested the association of gut microbiota with MMD. Fecal samples were collected from 27 patients with MMD, 7 patients with non-moyamoya intracranial large artery disease (ICAD) and 15 control individuals with other disorders, and 16S rRNA were sequenced. Although there was no difference in alpha diversity or beta diversity between patients with MMD and controls, the cladogram showed *Streptococcaceae* was enriched in patient samples. The relative abundance analysis demonstrated that 23 species were differentially abundant between patients with MMD and controls. Among them, increased abundance of *Ruminococcus gnavus* > 0.003 and decreased abundance of *Roseburia inulinivorans* < 0.002 were associated with higher risks of MMD (odds ratio 9.6, *P* = 0.0024; odds ratio 11.1, *P* = 0.0051). Also, *Ruminococcus gnavus* was more abundant and *Roseburia inulinivorans* was less abundant in patients with ICAD than controls (*P* = 0.046, *P* = 0.012). The relative abundance of *Ruminococcus gnavus* or *Roseburia inulinivorans* was not different between the p.R4810K mutant and wildtype. Our data demonstrated that gut microbiota was associated with both MMD and ICAD.

## Introduction

Moyamoya disease (MMD) is a progressive vasculopathy characterized by occlusive lesions in the terminal portion of the internal carotid arteries, which often causes ischemic complications^1–3^. Abnormal vascular networks called moyamoya vessels and periventricular anastomosis develops as collaterals, and fragile periventricular anastomoses, which create retrograde blood flow in the medullary arteries, are prone to bleed^4,5^.

The disease is rare, while it is endemic in East Asia. It has been shown that *RNF213* (*Mysterin*) on chromosome 17q25.3 is a susceptibility gene for MMD through a pedigree analysis of familial MMD^6,7^, and the p.R4810K founder mutation has been reported to increase the risk of MMD by > 300 times^6^. On the other hand, the penetrance of the mutation is low, and less than 1% of the mutation carriers develop MMD^8^, suggesting that other factors could trigger the development of the disease possibly by acting on this genetic predisposition^9^. Although viral infection has been postulated to be a potential risk factor for MMD, association between vasculopathy-related viruses and MMD has not been shown^10^, and thus, major non-genetic factors remain to be elucidated.

Microbiome, which forms the microbial communities inhabiting the human body, plays so many important roles in modulating our immune systems or promoting our metabolic operations^11^. They are composed of an enormous number of microbes that are both helpful and potentially harmful^12^. Most of them are symbiotic and some are pathogenic^13,14^. In a healthy body, symbiotic and pathogenic microbes coexist without any problems. However, the imbalance of gut microbiome has been reported in various disorders such as diabetes^15^, hypertension^16^, infection and inflammatory diseases^17^, intracranial aneurysm^18^, and mental disorders^19^. So far, the association between gut microbiome and MMD has hardly been evaluated and a comprehensive understanding of the microbiome-associated MMD pathology is necessary.

In this study, we investigated the relationship between intestinal bacteria and MMD by performing analysis of 16S ribosomal RNA (rRNA) in feces for case and control groups.

## Results

### Clinical characteristics

Clinical characteristics of the study population are shown in Table 1, including 27 patients with MMD (21 females and 6 males), 7 patients with non-moyamoya intracranial large artery disease (ICAD) (2 females and 5 males), and 15 controls (5 females and 10 males) with other disorders who were confirmed not having MMD or ICAD. Proportion of females was higher in patients with MMD than controls (*P* = 0.012). The median age (interquartile range) was 33 (37) years for patients with MMD, 45 (15) for patients with ICAD, and 57 (23) for controls. Both patients with MMD and patients with ICAD were significantly younger than control individuals (*P* = 0.0011 and *P* = 0.040). The p.R4810K mutation was detected in 77.7% in patients with MMD, 42.9% in patients with ICAD, and none in controls. Vascular risk factors such as hypertension, diabetes mellitus, dyslipidemia, and habits of smoking or drinking were not significantly different between patients with MMD or ICAD and controls. History of antibiotic use was higher in patients with MMD than controls (*P* = 0.042).

**Table 1:** Characteristics of the Study Population.

	Control	MMD	*P* value (MMD vs control)	ICAD	*P* value (ICAD vs control)	All CVD (MMD + ICAD)	*P* value (All CVD vs control)
Number, n	15	27		7		34	
Female, n (%)	5 (33.3)	21 (77.8)	0.012	2 (28.5)	1	23 (67.6)	0.054
Age, median (IQR)	57 (23)	33 (37)	0.0011	45 (15)	0.040	37.5 (28.75)	< 0.001
History of antibiotics use, n (%)	2 (13.3)	13 (48.1)	0.042	0 (0)	1	13 (38.2)	0.10
Family history of MMD, n (%)	0 (0)	9 (33.3)	0.016	0 (0)	1	9 (26.5)	0.042
Hypertension, n (%)	6 (40.0)	5 (18.5)	0.16	2 (28.6)	1	7 (20.6)	0.18
Diabetes, n (%)	3 (20.0)	3 (11.1)	0.65	2 (28.6)	1	5 (14.7)	0.69
Dyslipidemia, n (%)	3 (20.0)	6 (22.2)	1	4 (57.1)	0.15	10 (29.4)	0.73
Coronary artery disease, n (%)	2 (13.3)	1 (3.7)	0.29	0 (0)	1	1 (2.9)	0.22
Current drinker, n (%)	5 (33.3)	6 (22.2)	0.24	1 (14.3)	0.32	7 (20.6)	0.47
Current smoker, n (%)	2 (13.3)	2 (7.4)	0.61	1 (14.3)	1	3 (8.8)	0.64
Genotype, n			< 0.001		0.023		< 0.001
Homozygote	0	1		0		1	
Heterozygote	0	21		3		24	
Wildtype	15	5		4		9	
Age at onset, median (IQR)	NA	22 (33)		39 (15)		33 (37.25)	

MMD represents moyamoya disease; ICAD, non-moyamoya intracranial large artery disease; CVD, cerebrovascular disease; NA, not applicable; IQR, interquartile range. All CVD consists of MMD and ICAD.

### Absence of dysbiosis in moyamoya disease

**Figure 1:**
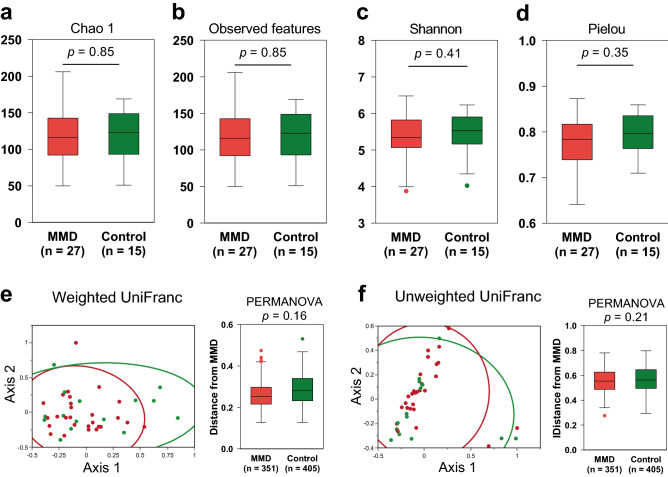
Alpha and beta diversity between patients with moyamoya disease (MMD) and control subjects. (**a-d**) Comparison of alpha diversity between patients with MMD and control subjects based on the Chao1, observed, Shannon, and Pielou indices. No significant difference was found in alpha diversity based on the Chao1 (**a**), observed features (**b**), Shannon (**c**) and Pielou (**d**) (*P* > 0.05 by Mann–Whitney *U* test). Box shows interquartile range and median and whisker indicates upper and lower 95% ranges. (**e**) 2D Principal Coordinate Analysis plots of beta diversity of control subjects (green dots) and patients with MMD (red dots), and the group difference tested by PERMANOVA showed no significant difference. Ellipses indicate 95% confidence intervals. (**f**) Group difference plots in beta diversity between MMD patients and control subjects. The distances between each sample in MMD group and each other sample are shown, and n means a total number of measured distances.

We first compared the diversity of gut microbiota between patients with MMD and controls. As shown in Fig. 1a–d, there was no significant difference in alpha diversity (diversity within a single sample), which includes species richness (Chao1 and observed features), richness and evenness (Shannon index) and evenness (Pielou index) between them. Principal coordinate analysis of weighted and unweighted UniFrac distances showed no significant difference in beta diversity (partitioning of diversity among communities) between patients with MMD and controls, either (*P* > 0.05 by PERMANOVA; Fig. 1e–f). We also performed stratified analysis. Age group 30–69 or male specific analyses did not show significant difference in either alpha or beta diversity (Supplemental Figs. 1–2). Although female specific analysis showed significant differences in beta diversity and metrics of richness but not evenness in alpha diversity (Supplemental Fig. 3), this may be because the diversity of the control samples was underestimated due to the small sample size (n = 5) as compared with cases (n = 21). We assume that the reduced diversity of gut microbiome (dysbiosis) is not likely to be associated with MMD. Additionally, we analyzed the difference of diversity between patients with MMD and those with ICAD (Supplemental Fig. 4). There was no significant difference in either alpha or beta diversity.

**Figure 2:**
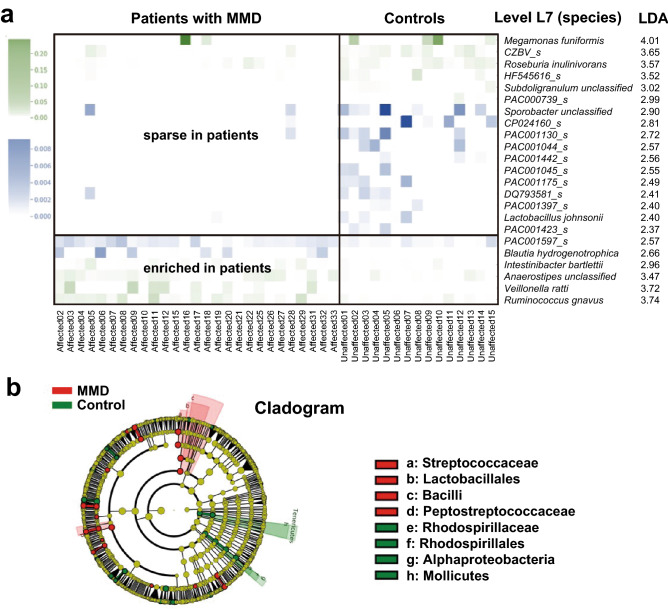
Relative abundance analysis of gut microbiota. (**a**) The LEfSe analysis showed several microbial taxa that were significantly different between patients with MMD and control individuals. The discriminative taxa with top ranked LDA scores at the species level (L7) are shown. Heatmap demonstrates distinct patterns between patients with MMD and controls. Blue represents microbes with lower relative abundance (mostly < 0.001 as shown in the scale bar in the left panel), and green represents microbes with relative abundance higher than the blue ones. (**b**) The cladogram of the discriminative taxa identified by LEfSe was shown. *Streptococcus* of the *Firmicutes Bacilli* class, and *Lactobacillales* were enriched in patients with MMD as compared with controls, whereas *Alphaproteobacteria* of the *Proteobacteria* division, *Rhodospirillaceae,* and *Mollicutes* were enriched in controls.

**Table 2:** Differential abundance analysis using DESeq2 between patients with MMD and controls.

Species enriched in patients with MMD	Log_2_ Fold Change	*P* value	Adjusted *p* value
***Intestinibacter bartlettii***	5.83	0.00014	0.0080
*Sellimonas intestinalis*	7.98	0.00077	0.022
***Ruminococcus gnavus***	3.47	0.0025	0.048
*Clostridium ramosum*	3.81	0.0069	0.098
*Bacteroides fragilis*	5.14	0.016	0.13
*Streptococcus* unclassified	0.70	0.17	0.62

Species sparse in patients with MMD	Log_2_ Fold Change	*P* value	Adjusted *p* value
*Parabacteroides* unclassified	− 3.64	0.011	0.12
*Alistipes onderdonkii*	− 5.15	0.013	0.12
*Bacteroides caccae*	− 6.18	0.018	0.13
*CZBV_s*	− 4.93	0.025	0.16
*Bifidobacterium* unclassified	− 2.44	0.094	0.53
*Roseburia inulinivorans*	− 2.76	0.11	0.59

Microbial taxa shown to be associated with MMD in both LEfSe and DESeq2 analyses are shown in bold.

**Figure 3:**
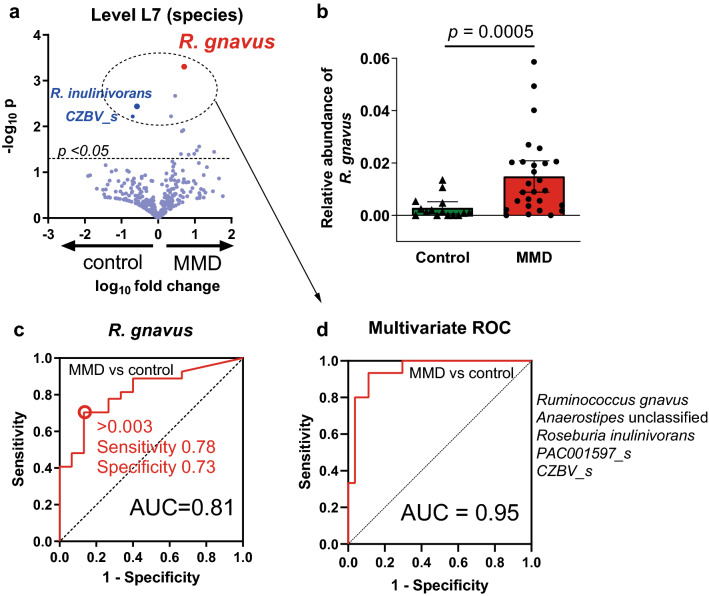
Association of *R. gnavus* with MMD. (**a**) The volcano plot showed *R. gnavus* had the lowest *p* value. (**b**)  The relative abundance of R. gnavus was higher in MMD than in controls. Bars and whiskers indicate means and standard deviations. (**c-d**) Univariate (**c**) and multivariate (**d**) Receiver Operating Characteristics (ROC) analysis indicated that the relative abundance of *R. gnavus* has good discrimination capacity for MMD.

**Table 3:** Univariate and multivariable analysis to test the association of the high relative abundance of *R. gnavus* > 0.003 with MMD as compare to the control.

Univariate analysis		
	Odds ratio (95% CI)	*P*
*R. gnavus*	9.62 (2.23–41.46)	0.0024
Age	0.95 (0.91–0.98)	0.0055
Sex	7.00 (1.72–28.54)	0.0070
Antibiotic use	6.04 (1.14–32.04)	0.035

**Multivariable analysis**		
**Model 1**	Odds ratio (95% CI)	*P*
*R. gnavus*	27.32 (2.00–337.14)	0.013
Age	0.94 (0.88–0.99)	0.048
Sex	21.43 (1.80–256.55)	0.016

**Model 2**	OR (95% CI)	*P*
*R. gnavus*	16.76 (2.73–102.84)	0.0023
Antibiotic use	12.29 (1.52-99.22)	0.019

In multivariable analysis, association of the relative abundance of *Ruminococcus gnavus* (*R. gnavus*) with moyamoya disease (MMD) was tested with adjustment for age and sex (model 1), with adjustment for history of antibiotics use (model 2). The p.R4810K mutation was not included because none of the controls have the mutation (odds ratio should be infinite).

**Figure 4:**
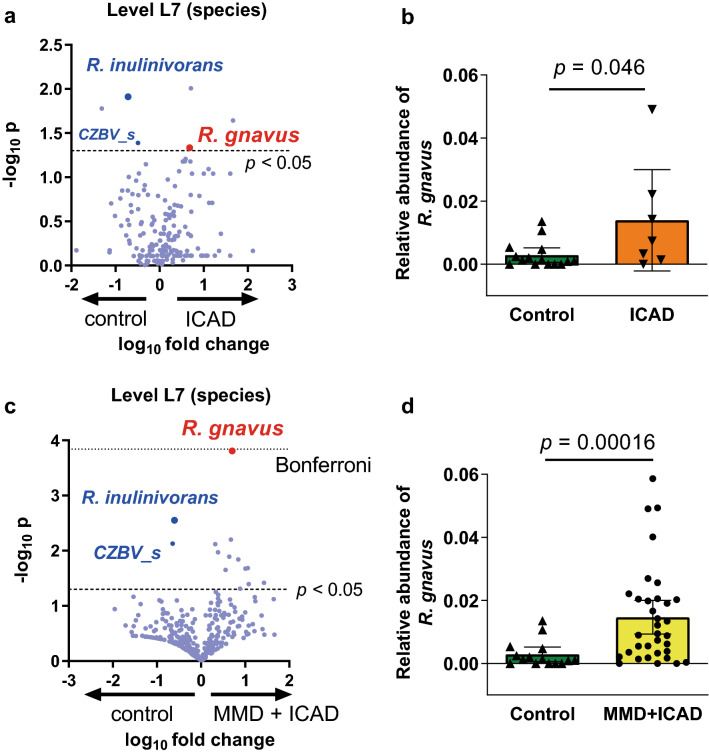
Association of *R. gnavus* with non-moyamoya intracranial large artery disease (ICAD). (**a**) The volcano plot showed *R. gnavus* was one of the top ranked microbes that was abundant in patients with ICAD as compared with controls. (**b**) Relative abundance of *R. gnavus* was significantly associated with ICAD (*P* = 0.046). Bars and whiskers indicate means and standard deviations. (**c,d**) When combining patients with MMD and those with ICAD, the volcano plot shows that the relative abundance of *R. gnavus* was significantly higher in patients than controls, and the *p* value was close to the Bonferroni corrected threshold (fine dotted line). Decreased abundance of *R. inulinivorans* and *CZBV_s* was observed not only in MMD (Fig. 3a) but also in ICAD (**a**) or in cerebrovascular disease (MMD + ICAD) (**c**).

### Differential abundance of gut microbiota in moyamoya disease

Next, we performed relative abundance analysis to identify specific microbes that are associated with the disease. Linear discriminant analysis effect size (LEfSe) showed that the relative abundance was significantly different between patients with MMD and controls in several microbes at the species level (level 7 = L7) (Fig. 2a). Six species including *Ruminococcus gnavus* (*R. gnavus*), *Veillonella ratti* (*V. ratti*), and *Intestinibacter bartlettii* (*I. bartlettii*) were enriched in patients with MMD and 17 species including *Megamonas funiformis* (*M. funiformis*), *CZBV_s*, and *Roseburia inulinivorans* (*R. inulinivorans*) were sparse in those patients (Fig. 2a). Heatmap of the relative abundance of these species clearly showed the distinct distribution between patients with MMD and controls. As for 17 species which are sparse in patients with MMD and enriched in controls, an average z-score was -0.252 for patients with MMD and 0.453 for controls. As for 6 species which are enriched in patients with MMD and sparse in controls, an average z-score was 0.252 for patients with MMD and -0.453 for controls.

To control the difference of demographic and clinical characteristics between patients with MMD and controls, we additionally performed differential abundance analysis using DESeq2 with adjustment for such variables. As a result, *I. bartlettii*, *Sellimonas intestinalis*, and *R. gnavus* were shown to be significantly increased in patients with MMD than controls with an adjusted *p* value of < 0.05 (Table 2). Thus, significant association of *R. gnavus* and *I. bartlettii* in LEfSe analysis has been replicated. Although some of the taxa in LEfSe analysis did not reach statistical significance in DESeq2 analysis, there was a large overlap between the analyses, e.g., *CZVB_s* and *R. inulinivorans* were listed as high ranked taxa that were decreased in patients with MMD. We further confirmed the result by another program, metagenomeSeq (Supplemental Table 1), showing that most high ranked taxa were overlapped with those listed in DESeq2 analysis. It often happens that different analyses produce discrepant results^20^.

Cladogram showed *Streptococcaceae* (family, L5) and *Peptostreptococcaceae* (L5) were enriched in patient samples, while *Rhodospirillaceae* (L5)*, Alphaproteobacteria* (class, L3)*,* and *Mollicutes* (L3) were sparse in patients with MMD (Fig. 2b). Among them, *Streptococcus* (genus, L6) in *Streptococcaceae* was shown to be associated with MMD in LEfSe analysis.

### Association of *R. gnavus* with MMD

We further analyzed *R. gnavus* because it showed the highest linear discriminant analysis (LDA) score of 3.74 among species that were enriched in patients with MMD (Fig. 2a) and because significant association of *R. gnavus* with MMD was confirmed by multivariable adjusted differential abundance analysis by DESeq2. The volcano plot confirmed that the relative abundance of *R. gnavus* was significantly higher in patients with MMD (mean ± standard deviation of 0.015 ± 0.015) than in controls (0.0028 ± 0.0041) with the lowest *p* value of 0.00050 (Fig. 3a,b, Supplemental Table 2). Receiver Operating Characteristic (ROC) analysis demonstrated that the relative abundance of *R. gnavus* was an effective biomarker of MMD with an area under the curve (AUC) of 0.81 (Fig. 3c). With an optimal cutoff value of 0.003, the sensitivity was 0.78 and the specificity was 0.73. Multivariable analysis incorporating all species with a *p* value of < 0.01 (dotted circle in Fig. 3a, listed in Supplemental Table 2) increased the accuracy of differentiating MMD from the control, with the AUC of 0.95 (Fig. 3d). Supervised analysis by Random Forest Classifier confirmed the effectiveness of the biomarker with the AUC of 0.69 for univariate analysis and 0.86 for multivariable analysis (Supplemental Fig. 5a–b).

**Figure 5:**
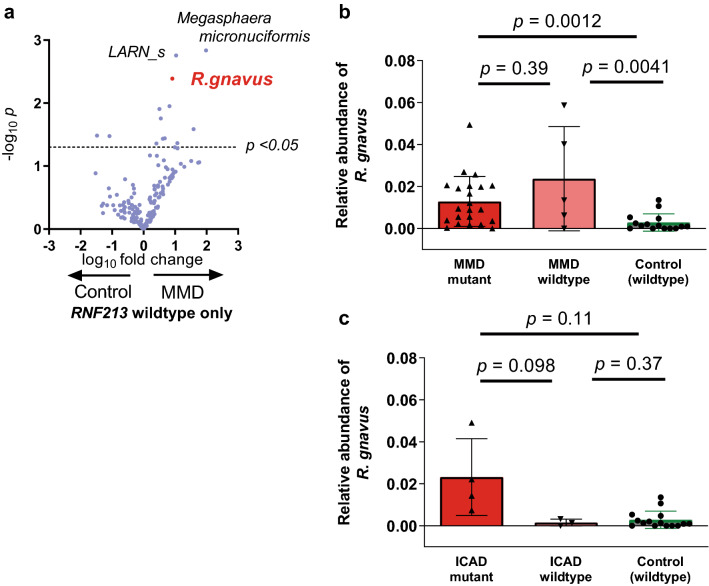
Stratification analysis of the *RNF213* p.R4810K mutation to assess the effect of *R. gnavus* on MMD and ICAD. (**a**) To minimize the effect of the p.R4810K mutation in *RNF213* gene, wildtype-specific analysis was performed. *R. gnavus* showed the third lowest *p* value as shown in the volcano plot. (**b**) There was no significant difference between *RNF213* mutant and wildtype in the abundance of *R. gnavus* in patients with MMD (*P* = 0.39). Bars and whiskers indicate means and standard deviations. (**c**) In patients with ICAD, those having the p.R4810K mutation showed a trend for higher relative abundance of *R. gnavus* than those without the mutation, but it was not statistically significant (*P* = 0.098).

According to the univariate logistic regression analysis (Table 3), the relative abundance of *R. gnavus* of > 0.003 was associated with 9.62 (95% confidence interval [CI], 2.23–41.46, *P* = 0.0024) times higher risk of MMD. It remained significant in multivariable analysis adjusted for age and sex (odds ratio, 27.32; 95%CI, 2.00–337.14; *P* = 0.013 for Wald test; Table 3) or for history of antibiotics use (odds ratio, 16.76; 95%CI, 2.73–102.84; *P* = 0.0023). Adjustment for age, sex and history of antibiotics was unreliable due to the shortage of statistical power. Because none of the control individuals have the p.R4810K mutation, adjustment for the mutation could not be reliably tested. Significant association was confirmed in age-matched analysis between 12 patients with MMD and 12 controls (*P* = 0.0025, Supplemental Fig. 6a) and in age- and sex-matched analysis (*P* = 0.032, Supplemental Fig. 6b). Although we collected fecal samples at least 4 months after the last antibiotics usage (median, 6.4 month; interquartile range, 93.0 month) to avoid its effect on microbiome, we compared the relative abundance of *R. gnavus* between those who had a history of antibiotics use and those who did not because it potentially affects the relative abundance of the microbiome. The result showed that history of antibiotics use did not significantly affect the relative abundance of *R. gnavus* (Supplemental Fig. 7), although antibiotics use in patients with MMD showed a trend to decrease the relative abundance of *R. gnavus*. Considering that history of antibiotics use was more frequent in patients with MMD, the association of *R. gnavus* and MMD can be underestimated rather than be overestimated.

**Figure 6:**
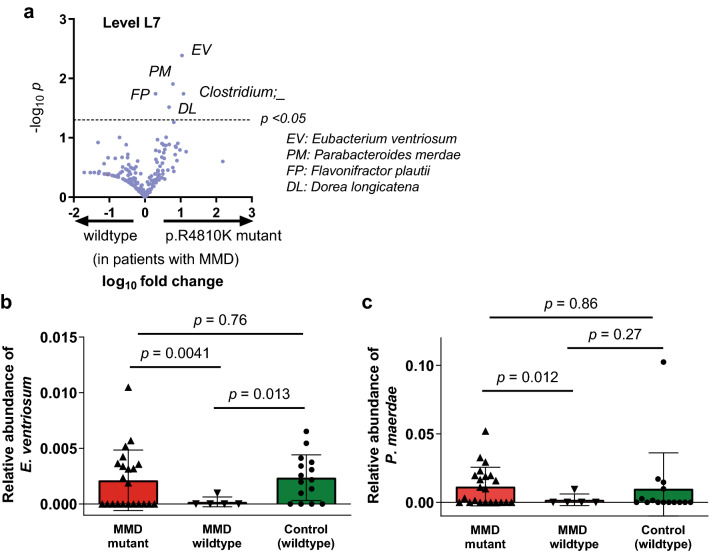
Relative abundance analysis of gut microbiota according to the p.R4810K mutation. (**a**) Relative abundances of gut microbiota were compared between MMD patients with the p.R4810K mutation and those without it. *Eubacterium ventriosum* (*E. ventriosum*) showed the lowest *p* value, followed by *Parabacteroides merdae* (*P. merdae*), *Clostridium*, *Flavonifractor plautii*, and *Dorea longicatena*. (**b-c**) Relative abundance of *E. ventriosum* (**b**) and that of *P. merdae* (**c**) was compared among MMD patients with the p.R4810K mutation (homozygotes and heterozygotes), MMD patients without the mutation, and control individuals (without the mutation). In either analysis, there was no significant difference between patients with the p.R4810K mutation and control individuals. Bars and whiskers indicate means and standard deviations.

### Association of *R. gnavus* with ICAD and combined analysis with MMD

We also tested the association of *R. gnavus* with ICAD, and the microbe was significantly more abundant in patients with ICAD as compared with controls (*P* = 0.046; Fig. 4a–b). Next, we compared the difference between MMD and ICAD. There was no difference in the relative abundance of *R. gnavus* between them (Supplemental Table 3). Then we combine MMD and ICAD as cerebrovascular disease. The volcano plot of differentially abundant microbes between patients with cerebrovascular disease (MMD + ICAD) and controls demonstrated that *R. gnavus* had the lowest *p* value (Fig. 4c,d, *P* = 0.00016), which was nearly the Bonferroni corrected threshold (Fig. 4c, fine dotted line, *P* = 0.00014). We further performed regression analyses to test the association of the relative abundance of *R. gnavus* with cerebrovascular disease, and significant association was shown in both univariate and multivariable analyses (Supplemental Table 4).

We also performed age- and sex-matched analysis. The relative abundance of *R. gnavus* was significantly higher in patients with MMD and patients with cerebrovascular disease (MMD + ICAD) than controls (*P* = 0.032 and *P* = 0.020, Supplemental Fig. 4b), although the difference between ICAD and control did not reach statistical significance (*P* = 0.10). These results suggest that *R. gnavus* is associated not only with MMD but also with ICAD.

### Interaction between the *RNF213* p.R4810K mutation and *R. gnavus* in MMD and ICAD

To minimize the chance of confounding with the *RNF213* p.R4810K mutation for the association of *R. gnavus* with MMD, we performed stratified analysis. Because all the control subjects have wildtype *RNF213*, we restricted the analysis only for those who have wildtype *RNF213*. As shown in the volcano plot in Fig. 5a, *R. gnavus* remains one of the top ranked microbes, showing differential abundance between patients with MMD and controls. In accordance with this, the relative abundance of *R. gnavus* was not different between patients with MMD having the *RNF213* p.R4810K mutation (homozygote and heterozygote) and those having the wildtype *RNF213* (Fig. 5b), while both of them showed significant differences from controls. In patients with ICAD, those having the p.R4810K mutation showed a trend for a higher relative abundance of *R. gnavus* than those without the mutation, but it was not statistically significant (*P* = 0.098, Fig. 5c). The sample size of 7 provides insufficient statistical power to detect the difference (α = 0.10, β = 0.51), and 21 cases with ICAD will be required.

Next, we analyzed the proportion of individuals who have both the relative abundance of *R. gnavus* > 0.003 and the p.R4810K mutation. Those who have both factors were seen in 63.0% of MMD, 42.9% of ICAD and none (0%) of the control group (Supplemental Table 5). On the other hand, those who have neither factor were seen in 3.7% of MMD, 28.6% of ICAD and 73.3% of the control group. Among patients with MMD and controls, the higher number of disease-related factors (1 point for the p.R4810K and 1 point for the relative abundance of* R. gnavus* >0.03) was associated with higher proportion of patients with MMD (8.3% for zero point, 69.2% for one point, and 100% for two points; *P* < 0.001 for Cochran-Armitage trend test).

We further analyzed the association of the relative abundance of *R. gnavus* and the p.R4810K mutation with clinical manifestation in MMD (Supplemental Table 6). Either *R. gnavus* or the p.R4810K mutation did not show significant association with bilateral involvement, childhood onset, or the severe form of onset (ischemic or hemorrhagic stroke). However, symptoms at onset were significantly different according to the number of risk factors (*P* = 0.043). Those who had two factors showed higher frequency of asymptomatic patients, whereas those who had one factor showed higher frequency of ischemic or hemorrhagic stroke. The number of factors was not related with the frequency of family history of MMD.

### Similarity of microbial changes between MMD and ICAD

Although comparison of microbiomes between MMD and ICAD is not the main objective of the study, the volcano plots of differentially abundant microbes between patients with MMD and controls (Fig. 3a) and those between patients with ICAD and controls (Fig. 4a) showed that there was overlap of top ranked microbes between them. Specifically, *R. gnavus* was increased and *R. inulinivorans* and *CZBV_s* were decreased both in patients with MMD and those with ICAD as compared with controls. Thus, we analyzed the association of *R. inulinivorans* with MMD and ICAD as was performed for *R. gnavus*. The relative abundance of *R. inulinivorans* was significantly lower in patients with MMD (*P* = 0.0037, supplemental Fig. 8a) and it was undetectable (relative abundance was zero) in 16 of 27 patients. ROC analysis showed that AUC was 0.79, and cutoff value of 0.002 rendered a sensitivity of 0.63 and specificity of 0.87 (supplemental Fig. 8b). Univariate logistic regression analysis showed that the relative abundance of *R. inulinivorans* of < 0.002 was associated with 11.05 (95% CI, 2.06–59.36, *P* = 0.0051) times higher risk of MMD. It remained significant after adjustment for antibiotic use, or for age and sex. Significant association of *R. inulinivorans* was also shown in patients with ICAD, and age- and sex-matched analysis confirmed the association of *R. inulinivorans* with ICAD and with cerebrovascular disease (MMD + ICAD) (Supplemental Fig. 8c–d). Then, association of the p.R4810K mutation with the relative abundance of *R. inulinivorans* was tested, and no significant difference was observed between mutant and wildtype either in MMD or ICAD (Supplemental Fig. 8e–f).

### Influence of *RNF213* p.R4810K mutation on gut microbe composition in MMD

Because it has been reported that RNF213 plays a role in defending the host from bacteria such as *Salmonella* by ubiquitinating lipopolysaccharides (LPS) on the surface of the microbes, we investigated the effect of the p.R4810K mutation on the composite of the gut microbiota. As shown in Fig. 6a, *Eubacterium ventriosum* (*E. ventriosum*), *Parabacteroides merdae* (*P. merdae*), *Clostridium*, *Flavonifractor plautii*, and *Dorea longicatena* were higher in its relative abundance in patients with MMD having the p.R4810K mutation (homozygote and heterozygote) as compared with those without the mutation. However, the relative abundances of *E. ventriosum* were comparable between patients having the p.R4810K mutation and control individuals (without the p.R4810K mutation) (*P* = 0.76, Fig. 6b), and it was significantly lower in MMD patients without the mutation than control individuals (*P* = 0.013), making it unlikely that the p.R4810K has direct effects on the relative abundance of *E. ventriosum.* The relative abundance of *P. merdae* was also comparable between patients with the mutation and control individuals (*P* = 0.86, Fig. 6c).

## Discussion

Although dysbiosis, defined as a reduction in microbial diversity, was not likely to be associated with MMD, the relative abundance was significantly different between patients with MMD and controls in several species of gut microbiota. According to the LDA score, *R. gnavus, I. bartlettii* or *V. ratti* was enriched, while *M. funiformis* or *R. inulinivorans* was sparse in patients with MMD as compared with controls. The cladogram showed that *Streptococcaceae* and *Peptostreptococcaceae* were enriched in patients with MMD, while *Rhodospirillaceae*, *Alphaproteobacteria*, and *Mollicutes* were sparse in those patients. It is interesting that increase of *Lachnospiraceae* (*R. gnavus* belongs to this family) and decrease of *Alphaproteobacteria* is caused by angiogenin^21^, which might be associated with upregulated angiogenesis in MMD^22^. Angiogenesis and vasculogenesis manifested in patients with MMD, e.g., basal moyamoya, periventricular anastomosis, and leptomeningeal anastomosis, are regarded as compensation of the stenosis of the main trunk of the internal carotid arteries. Leptomeningeal anastomosis can be observed in the contralateral side in patients with unilateral MMD^23^, suggesting that circulating factors are involved in the phenomenon. Such factors elevated in patients with MMD include transforming growth factor (TGF)-β1 and prostaglandin E2 (PGE2)^24,25^, which may possibly induce both fibromuscular thickening of the intima in the internal carotid artery and neovascular formation^26,27^. In this context, it is interesting that *Veillonella* or *Streptococcus* in the gut has been reported to be associated with an increased level of PGE2^28^. As discussed above, association of gut microbiome and proangiogenic cytokines seems to be bidirectional: proangiogenic cytokines, e.g., angiogenin, may form composition of gut flora, and at the same time, certain microbes, e.g., *Veillonella* or *Streptococcus,* may produce proinflammatory and proangiogenic conditions. Another interesting taxon is *M. funiformis*, which was significantly decreased in patients with MMD. Some articles reported that *M. funiformis* was a beneficial bacterium because it was decreased in the elderly^29^ and was enriched by a probiotic component of fermented tea^30^, whereas some reported that it is a harmful bacterium because it was associated with metabolic syndrome^31^. Intriguingly, the p.R4810K mutation, a risk factor of MMD and coronary artery disease, was inversely associated with diabetes mellitus^32,33^. Therefore, it may be possible that decrease of *M. funiformis* was positively associated with MMD and negatively associated with diabetes mellitus and metabolic syndrome.

We focused our analysis on *R. gnavus* as it was most significantly increased in patients with MMD at the species level. Both LEfSe analysis and DESeq2 analysis with adjustment of demographic and clinical characteristics showed that *R. gnavus* was significantly associated with MMD. ROC analysis showed the relative abundance of *R. gnavus* was effective in distinguishing MMD and the control, with the AUC of 0.81. Inclusion of other microbes that was associated with MMD increased the accuracy with the AUC of 0.97. Even when we restricted the analysis only to those without the p.R4810K mutation, the relative abundance of *R. gnavus* was significantly higher in patients with MMD than controls. Importantly, the risk of having MMD was as high as 9.6 times for individuals who have the relative abundance of *R. gnavus* of > 0.003. Any non-genetic factors that have such a large effect on MMD have not been reported to date, except for daily drinking which was associated with a higher risk of contralateral progression in unilateral MMD^9^. The positive association between *R. gnavus* and MMD will be worth to be confirmed in a larger independent study.

Our data also showed that an increased abundance of *R. gnavus* was significantly associated with ICAD e.g., middle cerebral artery occlusion. Considering that the p.R4810K confers the risk of both MMD and ICAD, it is not surprising that *R. gnavus* is also associated with both diseases. This is in line with the evidence that both *R. gnavus* and the p.R4810K mutation were associated with coronary artery disease^32,34^. Additionally, *R. gnavus* has been reported to be linked with systemic lupus erythematosus^35^ and Kawasaki disease^36^, which are causes of moyamoya syndrome. It is interesting that other microbes identified in our study such as *Streptococcus* and *Peptostreptococcus* were also associated with systemic lupus erythematosus^37^ and Kawasaki disease^38^. These lines of evidence suggest that proinflammatory condition induced by altered abundance of specific microbes may increase the risk of arterial diseases as well as immune-related diseases. In fact, *R. gnavus* produces glucorhamnan polysaccharide which activates dendritic cells to secrete TNF-α in a TLR4-dependent manner^39^. Both polysaccharide and TNF-α upregulates *RNF213*, a possible sensor of bacterial infections^40^. Dysfunction of RNF213 may cause insufficient elimination of pathogens, leading to prolonged inflammation.

It is noteworthy that there was substantial overlap of top ranked differentially abundant microbes between MMD and ICAD. Both MMD and ICAD were characterized by an increase of *R. gnavus* and a decrease of *R. inulinivorans* and CZBV_s, suggesting the possibility that these cerebrovascular diseases may share a common pathological background. It has previously been reported that the relative abundance of *R. inulinivorans* were decreased in men with low high-density lipoprotein (HDL) cholesterol than male controls^41^. This is in line with the evidence that HDL level was inversely associated with MMD^42^ and ICAD^43^. Other reports showed that *R. inulinivorans* was decreased in patients with atherosclerotic disease^44^ and those with Crohn’s disease^45^, suggesting that *R. inulinivorans* may be inversely associated with inflammatory condition.

Recently, the ubiquitylation of bacterial lipopolysaccharide by *RNF213* was reported^46^, and it restricts the proliferation of cytosolic *Salmonella*. Given that RNF213 plays a role in antibacterial defense, there appears a possibility that the p.R4810K mutation might affect the composite of gut microbiota. Thus, we tested whether the p.R4810K founder mutation was associated with microbial changes. Our data showed that the mutation was not associated with the relative abundance of *R. gnavus* in either patients with MMD or those with ICAD. Although the p.R4810K mutation showed a trend for higher abundance of *R. gnavus* in patients with ICAD, while there was no difference in patients with MMD, this would simply suggest that those who have neither the high relative abundance of *R. gnavus* nor the p.R4810K mutation are less likely to have MMD. In fact, those who have neither factor were seen in only 3.7% in MMD, while they were seen in 28.6% in ICAD. In relation to this, the higher number of disease-related factors, i.e., the relative abundance of *R. gnavus* > 0.003 and the p.R4810K mutation; 0–2 points), was associated with higher proportion of patients with MMD, and significant linear trend was shown, suggesting that these two factors fit an additive model. However, due to the small number of study population, the result needs to be confirmed in larger studies with additional tests for multiplicative models. Considering that 1–2% of the Japanese population have the p.R4810K mutation and 15 in 100,000 persons have MMD, less than 1% of those with the p.R4810K mutation develop MMD. Given that high relative abundance of *R. gnavus* is associated with 9.6 times higher risk of MMD, genotyping followed by 16S rRNA sequencing may become an option of screening examinations for early diagnosis of the disease. It will be worth testing whether the concept can be applied to the screening of family members of patients with MMD.

Although the relative abundance of *R. gnavus* or the p.R4810K itself did not show significant correlations with clinical manifestations of MMD including bilateral involvement, childhood onset, and severe form of onset (ischemic or hemorrhagic stroke), combination of the two factors showed significant correlation with the form of onset. Specifically, asymptomatic patients were more frequently seen in those who had both factors, whereas patients with ischemic or hemorrhagic stroke were more frequently seen in those who had one factor. This means that those who have the relative abundance of *R. gnavus* > 0.003 and the p.R4810K mutation have a higher chance of having MMD but they are more likely to be asymptomatic.

To further explore the effect of the p.R4810K mutation on the composition of microbiota, we compared the gut microbiota between MMD patients with the mutation and those without it. The relative abundances of *E. ventriosum* and *P. merdae* were found to be higher in MMD patients with the p.R4810K mutation. *E. ventriosum* was reported to be negatively associated with visceral fat accumulation^47^, and it was decreased in patients with acute coronary disease^48^. *P. merdae* may also work to protect against cardiovascular damage. Considering that the relative abundance of these microbes in patients with the p.R4810K mutation was comparable with that in control individuals, the difference observed between patients with and without the mutation is not likely to be a direct effect of the p.R4810K mutation. It would be rather reasonable to think that patients who have less genetic risk factors are more likely to have a larger number of non-genetic risk factors, e.g., decrease of protective gut microbiota. In fact, the relative abundance of *E. ventriosum* was significantly lower in MMD patients without the p.R4810K mutation than control individuals, suggesting that decrease of protective microbes such as *E. ventriosum* may also be associated with MMD, especially for patients who do not have the *RNF213* mutation.

There are several limitations in the present study. First, our findings have not been replicated in an independent cohort. However, association of *R. gnavus* was shown not only in MMD but ICAD, indicating a potential link between *R. gnavus* and cerebrovascular disease. Second, the number of study populations was small and the power of the statistical analyses was not sufficient, especially for the analysis of the effect of the p.R4810K mutation on the composition of gut microbiota. Besides, demographic and clinical characteristics were not well balanced among patients with MMD, those with ICAD, and controls. Although age- and sex-matched analysis confirmed the positive association of *R. gnavus* with MMD, replication studies are needed to confirm the results of the present study. Third, causality of *R. gnavus* and other microbes on the development of MMD remains unknown. Functional studies are needed to show the effect of *R. gnavus* and its metabolites on vascular homeostasis. Regardless of the causality of the gut microbes and MMD, our study at least showed that *R. gnavus* can be a biomarker of MMD. Last, we did not use shotgun metagenomic sequencing, which allows us to comprehensively capture all genes in all organisms present in a given complex sample including bacteria, fungi and viruses, making it possible to perform metagenome-wide association studies at the species level. It can also analyze their biological functional features. In this context, shotgun sequencing seems to be preferable, but it is costly, requires a machine resource and is more susceptible to contamination. Because this is a pilot study to test the association of gut microbiota and MMD, we selected 16S rRNA sequencing as a reasonable screening examination. Because we found a positive association of some gut microbes with MMD, it will be worthwhile performing shotgun sequencing to more deeply understand the relationship between gut microbiota and MMD.

## Conclusions

Our data showed that there was significant difference in 23 microbiome species in patients with MMD as compared with controls. Among them, increased abundance of *R. gnavus* (> 0.003) and decreased abundance of *R. inulinivorans* (< 0.002) were both associated with ~ 10 times higher risk of MMD. There was an overlap of top ranked differentially abundant microbes between MMD and ICAD, and increased abundance of *R. gnavus* and decreased abundance of *R. inulinivorans* were also significantly associated with ICAD. Functional analysis of these microbes will provide insight into the pathogenesis of MMD and ICAD and possibly help to develop new therapeutic options.

## Materials and methods

### Ethics approval and consent to participate

The study was approved by the IRB of Kyoto University (G1109) and all participants signed informed consent forms. Written informed consent was obtained from legal guardians if the patient was under the age of 20. All methods of this study were performed in accordance with relevant named guidelines and regulations.

### Study subjects

Patients with MMD were recruited from Kyoto University Hospital and collaborating hospitals. Those who were shown not to have occlusive cerebrovascular disease by MRI were selected as healthy controls. The diagnosis of MMD was based on the diagnostic criteria of the Japanese Research Committee on moyamoya disease of the Ministry of Health, Labour and Welfare, Japan^49,50^. ICAD was defined as stenoocclusive lesions in the major intracranial arteries including the internal cerebral artery, middle cerebral artery and anterior cerebral artery without fulfilling the diagnostic criteria of MMD, i.e., middle cerebral artery occlusion. Information on age, sex, age at onset, symptoms at onset, past medical history, history of antibiotics use, and family history of MMD was obtained either by interview or clinical chart review.

### Genotyping of the p.R4810K mutation in *RNF213*

Peripheral blood (2–10 ml) was collected from all participants. DNA was extracted from the blood samples using a QIAamp DNA Blood Mini Kit (Qiagen, Germantown, Maryland, USA) according to the manufacturer’s instructions. The quality and concentration of the extracted DNA were measured using an Infinite M200 PRO (TECAN, Kanagawa, Japan). The DNA was stored in a freezer at -30 °C until analysis. Genotyping of the p.R4810K mutation was conducted for all the participants using a TaqMan probe (Custom TaqMan SNP Genotyping Assays; Applied Biosystems, Foster City, CA, USA) and a 7300/7500 Real-Time PCR System (Applied Biosystems) according to the manufacturer’s instructions.

### Fecal collection and DNA extraction

Feces were obtained from participants and stored at − 80 °C until extraction. DNA was extracted from the fecal samples using NucleoSpin DNA Stool Kit (Takara Bio Inc., Shiga, Japan). 180–220 mg of defrosted samples was weighed in bead tubes, 850 µl first lysis buffer was added, and the suspension was shaken horizontally for a few seconds. Next, it was heated at 70 °C for 5 min, followed by a vortex on Vortex-Genie 2 (Scientific Industries, United States) at max speed for 10 min. The suspension was centrifuged at 13,000 × *g* for 3 min and 100 µl second lysis buffer was added to 600 µl of supernatant. The lysate was vortexed for 5 s and incubated at 4 °C for 5 min. After centrifugation at 13,000 × *g* for 3 min, the cleared lysate was obtained. The remaining steps, including wash and extraction, were performed according to the manufacturer’s protocol. Finally, DNAs were suspended in a 30 µl extraction buffer and stored immediately at -80 °C.

### Library preparation and sequencing of bacterial 16S rRNA

The concentration of DNA solution was quantified using SynergyLX (BioTek, United States) and QuantiFluor dsDNA System (Promega, United States). DNA was amplified using the 2-step tailed PCR to target the V3-V4 regions of bacterial 16S rRNA. 1st PCR was performed with the 1st-341f_MIX (5′-ACACTCTTTCCCTACACGACGCTCTTCCGATCT-NNNNN-CCTACGGGNGGCWGCAG-3′) and the 1st 805r_MIX (5′-GTGACTGGAGTTCAGACGTGTGCTCTTCCGATCT-NNNNN-GACTACHVGGGTATCTAATCC-3′) primers. Subsequently, 2nd PCR was performed with the 2ndF (5′-AATGATACGGCGACCACCGAGATCTACAC-Index2-ACACTCTTTCCCTACACGACGC-3′) and the 2ndR (5′- CAAGCAGAAGACGGCATACGAGAT-Index1-GTGACTGGAGTTCAGACGTGTG-3′) primers. Each PCR reaction was carried out using Ex Taq HS (Takara Bio Co., Shiga, Japan). V3-V4 PCR products were purified using AMPure magnetic beads (Beckman Coulter, United States) and quantified with Synergy H1 (BioTek, United States) and QuantiFluor dsDNA System (Promega, United States). Next, the PCR products were pooled to construct the sequencing library and the quality of the library was confirmed using Fragment Analyzer and dsDNA915 Reagent Kit (Advanced Analytical Technologies, United States). Sequencing was performed using the MiSeq Reagent Kit v3 (Illumina, United States) under the condition of 2 × 300 bp.

### Data analysis of 16S rRNA sequencing

Using the fastx_barcode_splitter tool of the FASTX -Toolkit (ver. 0.0.0.14), we extracted only the read sequences that exactly match the primer sequence used at the beginning of the read sequence. When the primer sequence contained N-mix, the procedure was repeated considering the number of N (6 types on the forward side × 6 types on the reverse side = 36 types). Primer sequences were deleted from the extracted reads using FASTX-Toolkit’s fastx_trimer.

Sequences with a quality value of less than 20 were then removed using sickle (ver. 1.33), and sequences with a length of 130 bases or less were discarded. Paired-end reads were joined using FLASH (ver. 1.2.11). After removing chimeric and noise sequences using the dada2 plugin of Qiime2 (ver. 2021.8), representative sequences and amplicon sequence variant tables were output. Taxonomic classification was performed by the feature-classifier plugin, and then alignment and phylogeny plugins were used to create the phylogenetic trees.

### LEfSE, DESeq2 and metagenomeSeq analyses

Operational taxonomic units (OTU) were analyzed, and bacterial community composition was compared using LEfSE at the genus (L6) and species (L7) levels. LEfSE (ver 1.0.8) was used to test whether there were strains with different relative abundances between groups^51^. Comparison of the relative abundance of gut microbiota was performed by Student’s *t* test with Welch’s correction.

Heatmap of discriminative taxa with top ranked LDA scores was made to show whether there are distinct patterns between patients with MMD and controls at L7 level. For quantitative evaluation of the heatmap, z-score was calculated for each taxon, and an average z-score in a specific block in the heatmap was then calculated.

Additionally, we used DESeq2 to identify species that are differentially abundant between patients with MMD and controls with controlling for age, sex, hypertension, diabetes mellitus, hyperlipidemia, use of antibiotics, and habits of smoking and drinking (everyday drinking of 1 or more units of alcohol)^52^. The raw count matrix of species was used as input. The species with a count of 30 or more in at least 15 samples were included in the analysis. Further, we used the fitZig function in metagenomeSeq to validate the results using zero-inflated gaussian mixture models^53^. The same covariates as DESeq2 were used to fit the model. *P*-values were controlled for false-discovery rate by Benjamini–Hochberg procedure, and species with adjusted *p*-values below 0.05 were considered differentially abundant between the phenotypes.

For LEfSe, we calculated relative abundances from the raw count matrix and used this as input. For DESeq2, the raw count matrix itself was used as input. DESeq2 calculates size factors correcting for library size and uses this to normalize the raw count internally.

### Evaluation of alpha and beta diversity

Alpha diversity and beta were analyzed using the diversity plugin of Qiime2. Alpha diversity represents diversity in a single sample. Among the indexes which evaluate alpha diversity, Chao 1 is a traditional index calculated according to the number of species observed, and it gives more weight to rare ones. Observed features evaluate the richness of species. Shannon is one of the most commonly used indexes and is calculated according to the proportion of species among all. If a large number of species are distributed equally, it gives a high Shannon index. Pielou's index also evaluates evenness, but it gives more weight to rare species. Beta diversity represents the difference of diversity between two samples. Unweighted Unifrac distance considers only the presence or the absence of a certain species, and weighted Unifrac distance is calculated according to the relative abundance of the species in the whole sample. Rarefaction analysis (Chao1, Shannon, etc.) and Principal Coordinate Analysis (PCoA) were performed. Alpha diversity was tested with Mann–Whitney’s *U* test. The statistical differences in beta diversity metrics between the groups was examined by permutational multivariate ANOVA (PERMANOVA). The test is based on the prior calculation of the distance from each patient within patients and to controls. For example, in the analyses shown in Fig. 1 e and f, the combinations of the two samples were _27_C_2_ = 27 × 26 / 2 = 351 in patients and 27 × 15 = 405 in controls. Stratified analysis for alpha and beta diversity was carried out. In addition to the sex specific analyses, age group between 30–69 were selected (top 10 percentile and bottom 10 percentile in controls were removed) because younger individuals were only seen in patients with MMD.

### Statistical analyses

Categorical variables were compared with the chi squared or Fisher’s exact tests where appropriate. In age-matched analysis, 12 pairs of patients with MMD and controls were selected within 10 years of age (Supplemental Fig. 3a). In age- and sex-matched analysis, 5 patients were selected from MMD, ICAD, and controls within 16 years of age (Supplemental Fig. 3b). ROC analysis was done to determine an optimal cutoff value for the relative abundance of *R. gnavus* in differentiating MMD from controls. The optimal cutoff on the ROC curve was defined as a point which maximizes the vertical distance from a diagonal line (Sensitivity = 1- Specificity). We performed univariate and multivariate logistic regression analyses to evaluate the associations of the relative abundance of *R. gnavus* with MMD. In multivariate analysis, age and sex were included in model 1, and history of antibiotics use were included in model 2. A Cochran–Armitage test was used to determine whether a linear trend exists between the number of associated factors and MMD. Pearson's correlation coefficient (*r*) was used to assess the linear correlation between continuous variables. These statistical analyses were carried out using the R version 4.0.5. ROC analysis, descriptions of volcano plots and heatmaps (seaborn clustermap) were done using the GraphPad Prism 9.3.1 and Python 3.8.13.

## Supplementary Information

Supplementary Information.

## Data Availability

The datasets generated and/or analyzed during the current study are available in the National Bioscience Database Center (NBDC) Human Database (http://humandbs.biosciencedbc.jp/en) with the accession number of JGAS000540.

## Abbreviations

MMD: Moyamoya disease
ICAD: Non-moyamoya intracranial large artery disease
OUT: Operational taxonomic units
LDA: Linear discriminant analysis
LEfSe: Linear discriminant analysis effect size
rRNA: Ribosomal RNA
ROC: Receiver Operating Characteristic
AUC: Area under the curve
*R. gnavus*: *Ruminococcus gnavus*
*R. inulinivorans*: *Roseburia inulinivorans*
*V. ratti*: *Veillonella ratti*
*I. bartlettii*: *Intestinibacter bartlettii*
*M. funiformis*: *Megamonas funiformis*
95%CI: 95% confidence interval
