# Supplementary material for: Increased abundance of Ruminococcus gnavus in gut microbiota is associated with moyamoya disease and non-moyamoya intracranial large artery disease
Source: Sci Rep. 2022 Nov 24;12:20244. doi: 10.1038/s41598-022-24496-9 (PMC9691692; doi:10.1038/s41598-022-24496-9)
Supplement: Supplementary file 1 — Supplementary Information. [file 41598_2022_24496_MOESM1_ESM.docx]

**Supplemental figure 1.** Stratified analysis of alpha and beta diversity in the population aged 30-69. They were compared between patients with moyamoya disease (MMD) and controls. There was no significant difference between the groups.


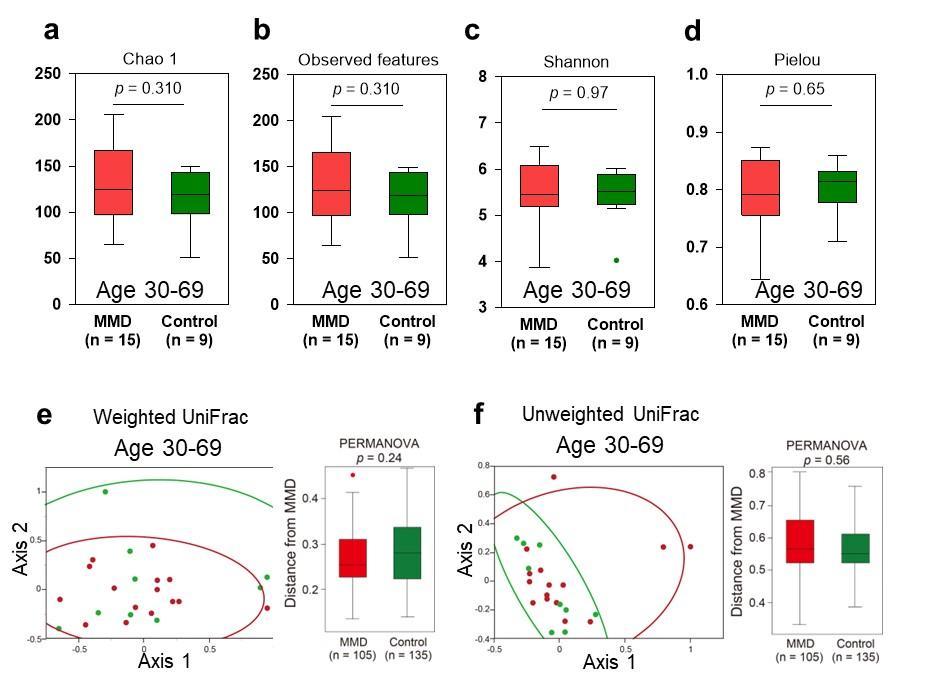


**Supplemental figure 2.** Male-specific analysis alpha and beta diversity between patients with moyamoya disease (MMD) and controls. There was no significant difference between the groups.

**
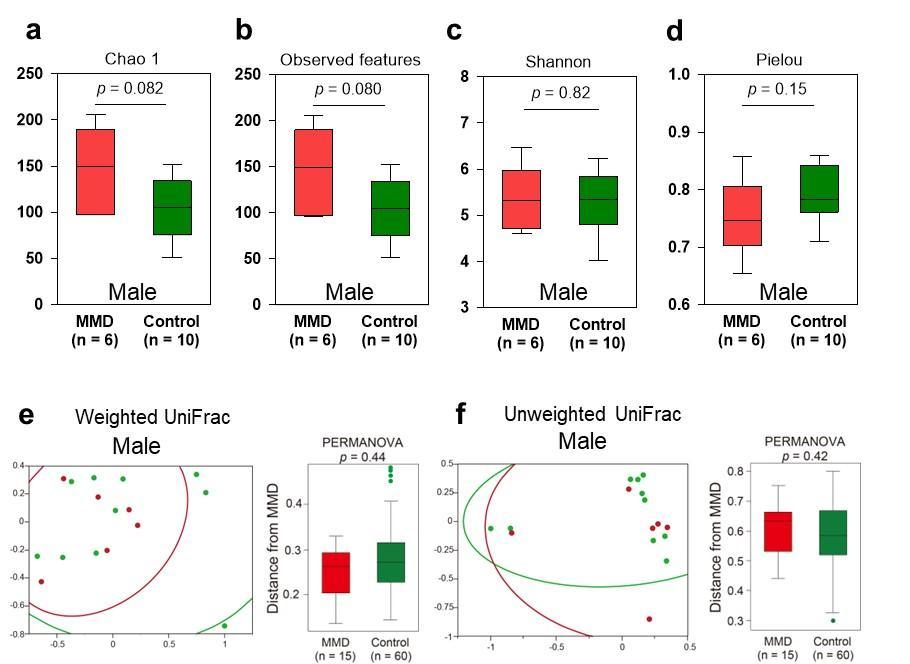
**

**Supplemental figure 3.** Female-specific analysis of alpha and beta diversity between patients with moyamoya disease (MMD) and controls. Chao1 and observed features in alpha diversity and weighted and unweighted UniFrac analyses showed significant differences between the groups. However, it should be noted that there are only 5 individuals in the control group, making the estimation unreliable.

**
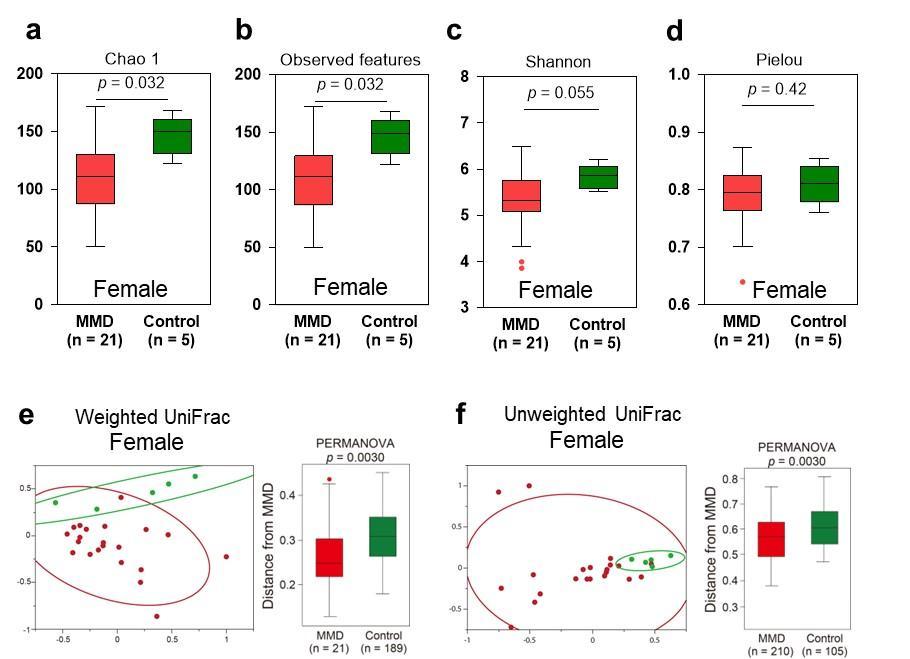
**

**Supplemental figure 4.** Alpha and beta diversity between patients with moyamoya disease (MMD) and patients with non-moyamoya intracranial large artery disease (ICAD). There was no significant difference between the groups.

**
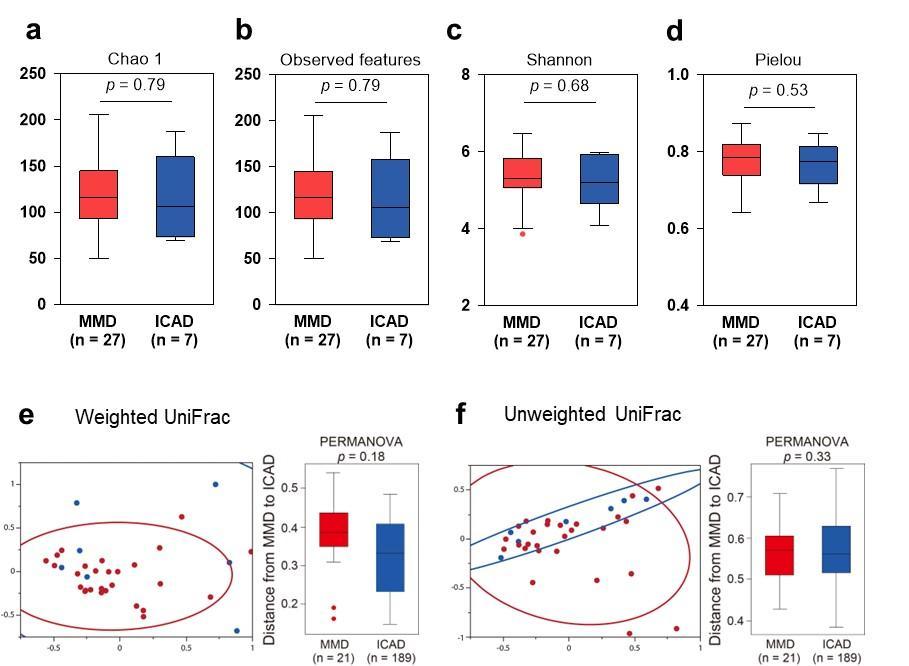
**

**Supplemental figure 5.** ROC curve for the Random Forest Classifier, showing that the relative abundance of *R. gnavus* distinguishes MMD from control with the AUC of 0.68. Inclusion of other taxa increased the accuracy with the AUC of 0.86.


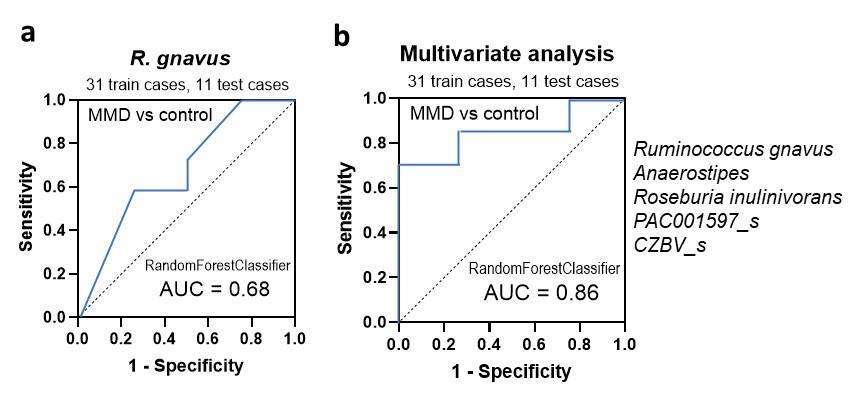


**Supplemental figure 6.** (**a**) Age-matched analysis comparing the relative abundance of *R. gnavus* between patients with moyamoya disease (MMD) and controls. The relative abundance was significantly higher in patients with MMD than in control individuals (*p* = 0.0025), where age did not differ between the groups (*p* = 0.70). (**b**) Age- and sex-matched analysis comparing the relative abundance of *R. gnavus* among patients with MMD, patients with non-moyamoya intracranial large artery disease (ICAD) and controls. Age and sex (M, male; F, female) of each group were shown in the bottom panel. The relative abundance of *R. gnavus* was significantly higher in patients with MMD than controls (*p* = 0.032). Although the difference between patients with ICAD and controls did not reach statistical significance (*p* = 0.10), when we combine ICAD and MMD, there was a significant difference (*p* = 0.020) as compared with controls.

**
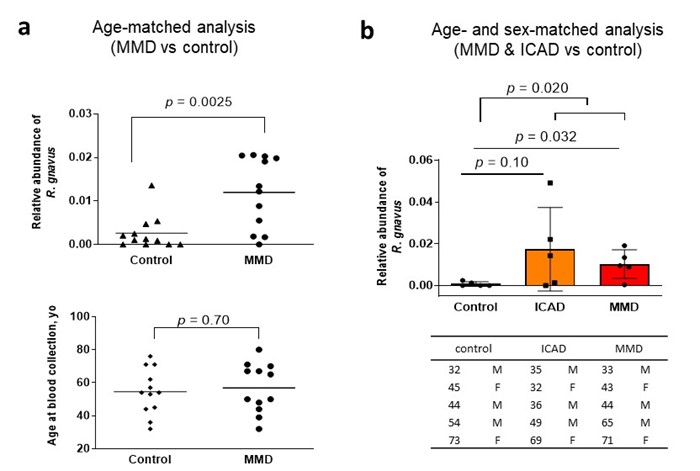
**

**Supplemental figure 7.** History of antibiotic use and the relative abundance of *R. gnavus*.

History of antibiotic use slightly reduced the relative abundance of *R. gnavus* in patients with MMD, although it was not statistically significant.


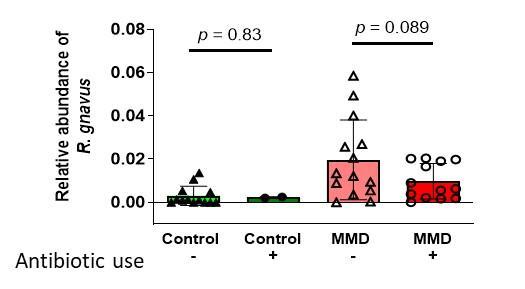


**Supplemental figure 8.** Association of *R. inulinivorans* with MMD and ICAD. (**a**) The relative abundance of *R. inulinivorans* was significantly lower in patients with MMD than controls. (**b**) Receiver Operating Characteristics (ROC) analysis indicated that the relative abundance of *R. inulinivorans* has good discrimination capacity for MMD with area under the curve (AUC) of 0.79. (**c**) The relative abundance of *R. inulinivorans* was significantly lower in patients with ICAD than controls. (**d**) Age- and sex-matched analysis showed the relative abundance of *R. inulinivorans* was significantly lower in patients with cerebrovascular disease (MMD + ICAD) than controls (*p* = 0.040). (**e-f**) There was no significant difference between mutant and wildtype (p.R4810K mutation in RNF213 gene) in the abundance of *R. inulinivorans* in patients with MMD (e) or in patients with ICAD (f).

**
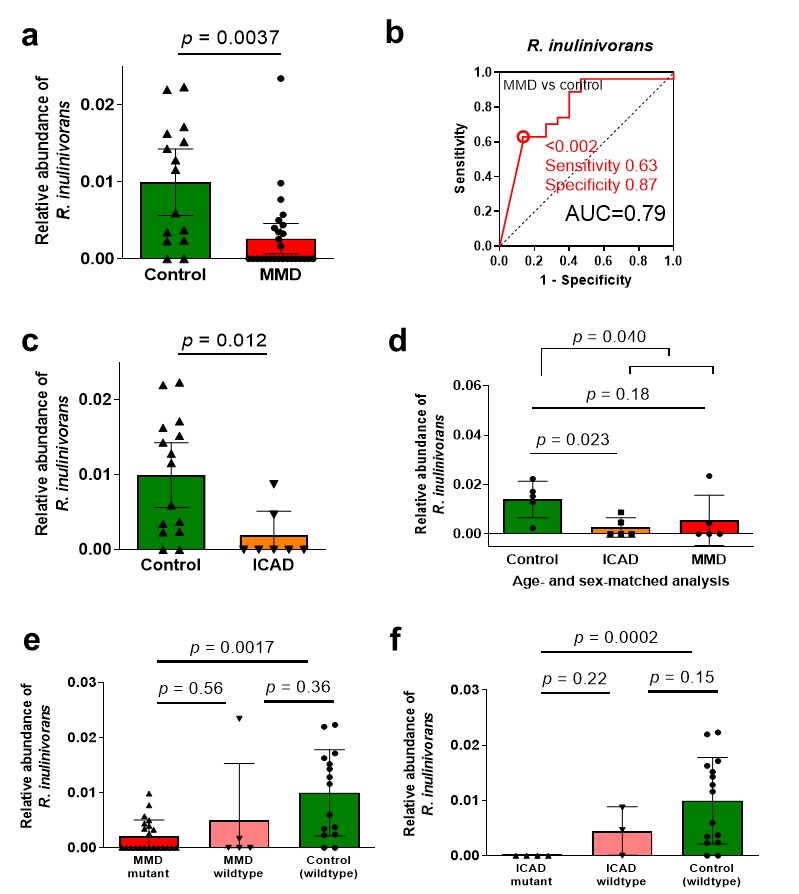
**

**Supplemental table 1.** Differential abundance analysis using metagenomeSeq between patients with MMD and controls.

| Species enriched in patients with MMD | Odds ratio (95% confidence interval) | *P* value | Adjusted *p* value |
| --- | --- | --- | --- |
| *Intestinibacter bartlettii* | 0.32 (0.066-1.4) | 0.022 | 0.29 |
| *Sellimonas intestinalis* | 0.40 (0.073-1.8) | 0.026 | 0.29 |
| *Ruminococcus gnavus* | 0.17 (0.014-1.2) | 0.031 | 0.29 |
| *Clostridium ramosum* | 0.35 (0.065-1.8) | 0.056 | 0.33 |
| *Streptococcus* unclassified | 0 (0-Infinite) | 0.070 | 0.33 |
| *Bacteroides fragilis* | 0.72 (0.16-3.1) | 0.070 | 0.33 |
| Species sparse in patients with MMD | Odds ratio (95% confidence interval) | *P* value | Adjusted *p* value |
| *Alistipes onderdonkii* | 2.1 (0.51-9.7) | 0.00044 | 0.025 |
| *Bacteroides caccae* | 2.0 (0.46-9.3) | 0.00097 | 0.028 |
| *CZBV_s* | 7.4 (1.6-43.4) | 0.0038 | 0.072 |
| *Roseburia inulinivorans* | 8.9 (1.6-97.3) | 0.035 | 0.29 |
| *Bacteroides xylanisolvens* | 1.3 (0.29-5.4) | 0.051 | 0.33 |
| *Roseburia* unclassified | 2.0 (0.38-13.6) | 0.068 | 0.33 |

**Supplemental table 2.** List of microbial taxa in volcano plot analysis between MMD and controls (Fig. 3a) with *p* value below 0.05.

|  | Log_10_ Fold Change | *P* value |
| --- | --- | --- |
| ***Ruminococcus gnavus*** | 0.71 | 0.00050 |
| ***Anaerostipes* unclassified** | 0.46 | 0.0022 |
| ***Roseburia inulinivorans*** | -0.58 | 0.0037 |
| ***PAC001597_s*** | 0.35 | 0.0060 |
| ***CZBV_s*** | -0.70 | 0.0061 |
| *LARN_s* | 0.69 | 0.012 |
| *Romboutsia timonensis* | 0.65 | 0.013 |
| ***Phocea massiliensis*** | 1.12 | 0.028 |
| *Eubacterium limosum* | 1.05 | 0.033 |
| *Akkermansia* unclassified | 1.53 | 0.036 |
| *Bifidobacterium bifidum* | 0.99 | 0.040 |
| *Bacteroides stercoris* | 0.62 | 0.040 |
| *Faecalimonas umbilicata* | 0.83 | 0.041 |

Species listed in the LEfSE analysis (Fig. 2a) were shown in bold.

**Supplemental Table 3.** Univariate analysis to test the difference of the relative abundance of *R. gnavus* and clinical variables between MMD and ICAD.

| **MMD vs ICAD** | | |
| --- | --- | --- |
| Univariate | Odds ratio (95% CI) | *P* |
| *R. gnavus* | 1.40 (0.21-9.12) | 0.73 |
| Age | 0.98 (0.94-1.02) | 0.32 |
| Sex | 8.75 (1.34-57.00) | 0.023 |
| p.R4810K | 5.87 (0.99-34.94) | 0.052 |

There was no significant association of the relative abundance of *Ruminococcus gnavus* (*R. gnavus*) with moyamoya disease (MMD) as compared to non-moyamoya intracranial large artery disease (ICAD). The p.R4810K mutation showed a trend to be associated with MMD as compared to ICAD, although statistically not significant (*p* = 0.052).

**Supplemental Table 4.** Univariate and multivariate analysis to test the association of the relative abundance of *R. gnavus* with cerebrovascular disease including MMD and ICAD.

| **All CVD (MMD + ICAD) vs control** | | |
| --- | --- | --- |
| **Univariate analysis** | | |
|  | Odds ratio (95% CI) | *P* |
| *R. gnavus* | 8.94 (2.22-35.95) | 0.002 |
| Age | 0.95 (0.91-0.98) | 0.005 |
| Sex | 4.18 (1.15-15.22) | 0.03 |
| Antibiotic use | 4.02 (0.78-20.78) | 0.096 |
| **Multivariate analysis** | | |
| **Model 1** | Odds ratio (95% CI) | *P* |
| *R. gnavus* | 40.17 (2.85-565.30) | 0.006 |
| Age | 0.93 (0.88-0.99) | 0.024 |
| Sex | 27.60 (1.95-390.98) | 0.014 |
| **Model 2** | OR (95% CI) | *P* |
| *R. gnavus* | 11.71 (2.54-53.88) | 0.0015 |
| Antibiotic use | 6.32 (0.97-41.08) | 0.054 |

In multivariate analysis, association of the relative abundance of *Ruminococcus gnavus* (*R. gnavus*) with moyamoya disease (MMD) was tested with adjustment for age and sex (model 1), with adjustment for history of antibiotics use (model 2). The p.R4810K mutation was not included because none of the controls have the mutation (odds ratio should be infinite).

**Supplemental Table 5.** Proportion of individuals who have the relative abundance of *R. gnavus* >0.003 and the p.R4810K mutation in MMD, ICAD and the control groups.

|  | *R. gnavus*>0.003 / p.R4810K | | |  |
| --- | --- | --- | --- | --- |
|  | 0 point | 1 point | 2 points | *P* |
| Phenotypes | |  |  | <0.001 |
| Control | 11 (73.3%) | 4 (26.7%) | 0 (0%) |  |
| ICAD | 2 (28.6%) | 2 (28.6%) | 3 (42.9%) |  |
| MMD | 1 (3.7%)  [8.3%] | 9 (33.3%)  [69.2%] | 17 (63.0%)  [100%] | [<0.01] |

Those who have the relative abundance of *R. gnavus* >0.003 take 1 point and those with the p.R4810K mutation (homozygote or heterozygote) take 1 point. If someone has both factors, they take 2 points. Difference among patients with MMD, those with ICAD, and controls were analyzed by Fisher’s exact test. Percentage in the [ ] indicates the proportion of patients among patients with MMD and controls, and the *p* value was calculated by Cochran-Armitage trend test.

**Supplemental Table 6.** Proportion of bilateral involvement and clinical symptoms at onset according to the relative abundance of *R. gnavus* and the p.R4810K mutation in patients with MMD.

|  | *R. gnavus* ≤0.003 | *R. gnavus* >0.003 | *P* | p.R4810K  wildtype | p.R4810K  mutant | *P* | *R. gnavus*>0.03 / p.R4810K | | | *P* |
| --- | --- | --- | --- | --- | --- | --- | --- | --- | --- | --- |
|  |  |  |  |  |  |  | 0 point | 1 point | 2 points |  |
| Number of patients | 6 | 21 |  | 5 | 22 |  | 1 | 9 | 17 |  |
| Angiographical distribution | | | 1 |  |  | 1 |  |  |  | 1 |
| Bilateral | 5 (83.3%) | 17 (81.0%) |  | 4 (80%) | 18 (81.8%) |  | 1 (100%) | 7 (87.5%) | 14 (82.4%) |  |
| Unilateral | 1 (16.7%) | 4 (19.0%) |  | 1 (20%) | 4 (18.2%) |  | 0 (0%) | 2 (12.5%) | 3 (17.6%) |  |
| Age at onset |  |  | 0.14 |  |  | 0.64 |  |  |  | 1 |
| Childhood (<17) | 0 (0%) | 8 (38.1%) |  | 3 (60%) | 10 (45.5%) |  | 0 (0%) | 4 (44.4%) | 9 (52.9%) |  |
| Adulthood | 6 (100%) | 13 (61.9%) |  | 2 (40%) | 12 (54.5%) |  | 1 (100%) | 5 (55.6%) | 8 (47.1%) |  |
| Symptoms at onset |  |  | 0.16 |  |  | 0.16 |  |  |  | 0.043 |
| Asymptomatic | 0 (0%) | 6 (28.6%) |  | 0 (0%) | 6 (27.3%) |  | 0 (0%) | 0 (0%) | 6 (35.3%) |  |
| Ischemic stroke | 1 (16.7%) | 2 (9.5%) |  | 1 (20.0%) | 1 (4.5%) |  | 0 (0%) | 3 (33.3%) | 0 (0%) |  |
| Hemorrhagic stroke | 1 (16.7%) | 0 (0%) |  | 1 (20.0%) | 1 (4.5%) |  | 0 (%) | 1 (11.1%) | 1 (5.8%) |  |
| TIA | 4 (66.7%) | 13 (61.9%) |  | 3 (60.0%) | 14 (63.6%) |  | 1 (100%) | 5 (55.5%) | 11 (64.7%) |  |
| Family history | 2 (33.3%) | 7 (33.3%) | 1 | 2 (40.0%) | 7 (31.8%) | 1 | 0 (0%) | 4 (44.4%) | 5 (29.4%) | 0.78 |

Those who have the relative abundance of *R. gnavus* >0.003 take 1 point and those with the p.R4810K mutation (homozygote or heterozygote) take 1 point. If someone has both factors, they have 2 points. TIA represents transient ischemic attack.
